# Supplementary material for: Analysis of genetic diversity among Chinese Cyclocybe chaxingu strains using ISSR and SRAP markers
Source: PeerJ. 2022 Sep 29;10:e14037. doi: 10.7717/peerj.14037 (PMC9527026; doi:10.7717/peerj.14037)
Supplement: Supplemental Information 1 [file peerj-10-14037-s003.docx]

**Supplementary Materials**

**Analysis of Genetic Diversity Among Chinese *Cyclocybe chaxingu* Strains Using Combined ISSR and SRAP Markers**

**Computer Code and Software**

**Code 1 Cluster analyses were performed based on the Jaccard similarity coefficient using the Unweighted Pair Group Method of Arithmetic Average (UPGMA) by R version 4.1.0**

**Code 2 Principal Coordinate Analysis (PCoA) was performed using R version 4.1.0**

**Code1:**

**#Cluster analyses were performed based on the Jaccard similarity coefficient using the Unweighted Pair Group Method of Arithmetic Average (UPGMA)**

**setwd("C:/data/")**

**library(cluster)**

**library(vegan)**

**raw <- read.csv("ISSR.csv",header = T,row.names = 1)**

**raw2 <- vegdist(raw,method = "jaccard")**

**hc2 <- hclust(raw2,method = "average")**

**#The co-phenetic correlation of the clustering to the data matrix was calculated**

**hc2.coph <- cophenetic(hc2)**

**cor(raw2,hc2.coph)**

**#Select the optimum contour width**

**plot(hc2$height,nrow(raw):2,type="S",main = "Fusion levels-Chord-UPGMA",**

**ylab="k(number of clusters)", xlab="h(node height)",col="grey")**

**text(hc2$height,nrow(raw):2,nrow(raw):2,col="red",cex=0.8)**

**asw <- numeric(nrow(raw))**

**for(k in 2:(nrow(raw)-1))**

**{sil <-silhouette(cutree(hc2,k=k),raw2)**

**asw[k] <- summary(sil)$avg.width}**

**k.value=which.max(asw)**

**plot(1:nrow(raw),asw,type="h",**

**main="Silhoutte-optimal number of clusters,UPGMA",**

**xlab="k(number of groups)",ylab="Average silhouette width")**

**axis(1, k.value,paste("optimum",k.value,A="\n"),**

**col="blue",font=2,col.axis="blue")**

**points(k.value,max(asw),pch=16,col="blue",cex=1.6)**

**cat("","Silhouette-optimal number of clusters k =",k.value,**

**"\n","with average silhouette width of",max(asw),"\n")**

**#Make group charts**

**cutg<-cutree(hc2,k=k.value)**

**DD<-silhouette(cutg,raw2)**

**DDE<-sortSilhouette(DD)**

**AAA<-sort(cutg)**

**rownames(DDE)<-row.names(raw)[attr(DDE,"iOrd")]**

**plot(DDE,main="Silhouette**

**plot-Chord-Ward",col=AAA+1,cex.names=0.8,nmax.lab=100)**

**#Rearrange the distance matrix**

**library(gclus)**

**library(RColorBrewer)**

**DD <-reorder.hclust(hc2, raw2)**

**dend<-as.dendrogram(DD)**

**or <- vegemite(raw,DD)**

**heatmap(t(raw[rev(or$species)]),Rowv=NA,**

**Colv=dend,col=c("white",brewer.pal(5,"Greens")),scale="none",margin=c(4,4),ylab="Loci**

**(weighted averagesof sample)",xlab="sample")**

**#The final clustering diagram was made, and the number of groups was K. value**

**source("hcoplot.R")**

**hcoplot(DD,raw2,k=k.value)**

**
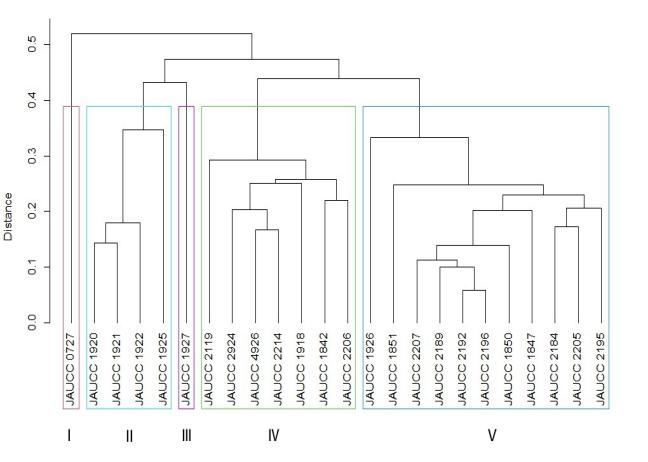
Figure 2 (a) UPGMA dendrogram of 24 *C.chaxingu* strains constructed using Jaccard distance analysis based on molecular profiles revealed by ISSR marker**

**Code2**

**# Principal Coordinate Analysis**

**setwd("C:/data/")**

**library(vegan)**

**library(ggplot2)**

**library(ggrepel)**

**library(ape)**

**df<-read.csv("ISSR.csv",header = T,row.names = 1)**

**jaccard_dist<-vegdist(df,method = "jac")**

**df.pcoa<-pcoa(jaccard_dist,correction = "none")**

**df.plot<-data.frame(df.pcoa$vectors)**

**x_label<-round(df.pcoa$values$Rel_corr_eig[1]*100,2)**

**y_label<-round(df.pcoa$values$Rel_corr_eig[2]*100,2)**

**ggplot(data=df.plot,aes(x=Axis.1,y=Axis.2))+**

**geom_point()+**

**geom_text_repel(label = rownames(df.plot)) +**

**theme_bw()+**

**theme(panel.grid = element_blank())+**

**geom_vline(xintercept = 0,lty="dashed")+**

**geom_hline(yintercept = 0,lty="dashed")+**

**labs(x=paste0("Dim1 ",x_label,"%"),**

**y=paste0("Dim2 ",y_label,"%"))**


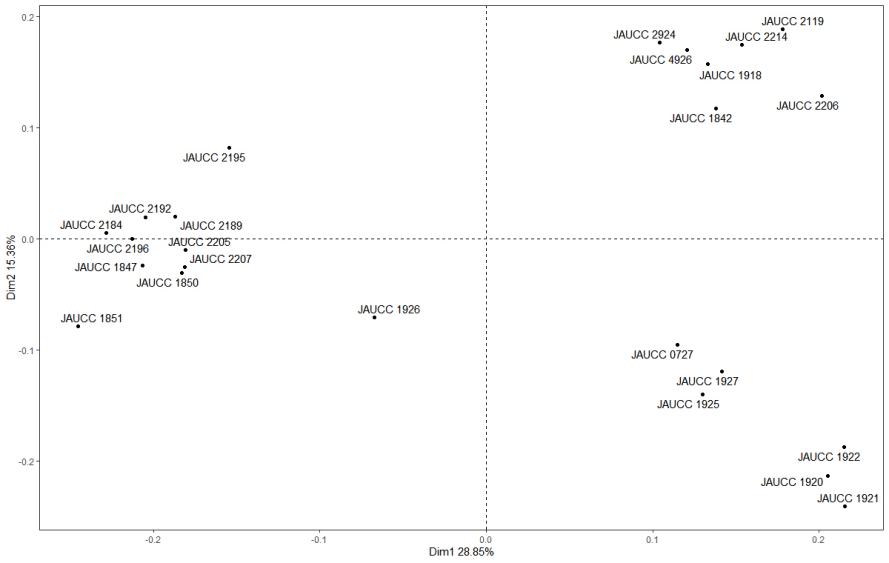
**Figure 2 (b) 2D principal component analysis (PCoA) based on genetic distance from ISSR data**
